# Cryptic lineage divergence in marine environments: genetic differentiation at multiple spatial and temporal scales in the widespread intertidal goby *Gobiosoma bosc*

**DOI:** 10.1002/ece3.3161

**Published:** 2017-06-22

**Authors:** Borja Milá, James L. Van Tassell, Jatziri A. Calderón, Lukas Rüber, Rafael Zardoya

**Affiliations:** ^1^ National Museum of Natural Sciences Spanish National Research Council (CSIC) Madrid Spain; ^2^ Department of Ichthyology American Museum of Natural History New York, NY 10024 USA; ^3^ Naturhistorisches Museum der Burgergemeinde Bern Bernastrasse 15, 3005 Bern Switzerland; ^4^ Institute of Ecology and Evolution University of Bern Baltzerstrasse 6, 3012 Bern Switzerland

**Keywords:** Florida, genetic diversity, genetic structure, Gobiidae, phylogeography, speciation, stabilizing selection

## Abstract

The adaptive radiation of the seven‐spined gobies (Gobiidae: Gobiosomatini) represents a classic example of how ecological specialization and larval retention can drive speciation through local adaptation. However, geographically widespread and phenotypically uniform species also do occur within Gobiosomatini. This lack of phenotypic variation across large geographic areas could be due to recent colonization, widespread gene flow, or stabilizing selection acting across environmental gradients. We use a phylogeographic approach to test these alternative hypotheses in the naked goby *Gobiosoma bosc*, a widespread and phenotypically invariable intertidal fish found along the Atlantic Coast of North America. Using DNA sequence from 218 individuals sampled at 15 localities, we document marked intraspecific genetic structure in mitochondrial and nuclear genes at three main geographic scales: (i) between Gulf of Mexico and Atlantic Coast, (ii) between the west coast of the Florida peninsula and adjacent Gulf of Mexico across the Apalachicola Bay, and (iii) at local scales of a few hundred kilometers. Clades on either side of Florida diverged about 8 million years ago, whereas some populations along the East Cost show divergent phylogroups that have differentiated within the last 200,000 years. The absence of noticeable phenotypic or ecological differentiation among lineages suggests the role of stabilizing selection on ancestral phenotypes, together with isolation in allopatry due to reduced dispersal and restricted gene flow, as the most likely explanation for their divergence. Haplotype phylogenies and spatial patterns of genetic diversity reveal frequent population bottlenecks followed by rapid population growth, particularly along the Gulf of Mexico. The magnitude of the genetic divergence among intraspecific lineages suggests the existence of cryptic species within *Gobiosoma* and indicates that modes of speciation can vary among lineages within Gobiidae.

## 1 INTRODUCTION

Recent research into diversification mechanisms in marine organisms using molecular data has revealed that pelagic larval stages, once thought to homogenize gene pools and prevent divergence, are often characterized by high levels of local recruitment (Barber, Palumbi, Erdmann, & Moosa, 2002; Jones, Milicich, Emslie, & Lunow, 1999; Swearer, Caselle, Lea, & Warner, 1999; Taylor & Hellberg, 2003), thus providing high potential for reduced gene flow and local adaptation (Bernardi, 2013). Therefore, although several studies have shown that fish population divergence can be consistent with geographic barriers (Rocha, Bass, Robertson, & Bowen, 2002; Shulman & Bermingham, 1995), an increasing number of studies are reporting results that are more consistent with the role of selection in driving speciation through local adaptation (Rocha & Bowen, 2008; Rocha, Robertson, Roman, & Bowen, 2005b; Taylor & Hellberg, 2005). For example, a number of studies in coral reefs have shown that once gene flow is restricted, niche specialization and sexual selection can readily cause divergence and speciation (Rocha et al., 2005b; Streelman, Alfaro, Westneat, Bellwood, & Karl, 2002; Taylor & Hellberg, 2005) even in the presence of gene flow (Crow, Munehara, & Bernardi, 2010; Puebla, Bermingham, & Guichard, 2012).

A well‐known example of the role of ecology in driving speciation in marine fishes is the radiation of the seven‐spined gobies (Gobiosomatini Birdsong, 1975), a New World tribe displaying great diversity in ecology, behavior, and color in its 26 genera and more than 130 species (Rüber, Van Tassell, & Zardoya, 2003; Van Tassell, 2012), and itself part of one of the most species‐rich teleost families (Gobiidae) with almost 2,000 species described to date (Agorreta et al., 2013; Thacker & Roje, 2011). The seven‐spined gobies are distributed in the western Atlantic and eastern Pacific throughout the coasts of America and achieve their highest species diversity in the Caribbean Sea (Van Tassell, 2012). While some species show rather restricted distributions (e.g., *Varicus lacerta* only found in Curaçao (Tornabene, Robertson, & Baldwin, 2016)), others are found along a wide latitudinal cline (e.g., *Aboma etheostoma* (Van Tassell, 2012). The seven‐spined gobies inhabit a range of habitats, from live corals and sponges to mudflats and sea grass beds (Böhlke & Chaplin, 1993), and exhibit a striking range of phenotypes and behaviors, including brightly colored species in coral reefs, species that specialize on removing ectoparasites from larger fish, or species that have evolved strict commensal associations with sponges, urchins, or shrimp (Böhlke & Robins, 1968). Molecular phylogenetic analyses have shown that the Gobiosomatini radiation has been driven largely by ecological, behavioral, and chromatic adaptations at different time scales (Rüber et al., 2003; Taylor & Hellberg, 2005).

However, even within groups like the seven‐spined gobies, characterized by highly ecologically specialized species assemblages at small geographic scales, some species stand out for showing no apparent phenotypic differentiation across large geographic ranges. Such is the case of the naked goby (*Gobiosoma bosc* Lacepède, 1800), a species found in shallow estuarine habitats, which shows what appears to be a complete lack of phenotypic differentiation across its large geographic distribution, which spans the Atlantic Coast of North America, from the state of New York in the North East, down through the Florida peninsula and along the Gulf Coast to the state of Texas. This apparent lack of geographically structured phenotypic variability can take place even at oceanic or continental scales and could be due to three main causes: (i) recent colonization across large areas, with insufficient time for lineage sorting and divergence; (ii) ongoing gene flow at regional or oceanic scales associated with pelagic larval stages, which may homogenize gene pools and prevent differentiation; and (iii) strong balancing selection on ancestral phenotypic traits despite local differences in ecological conditions.

In order to examine the validity of these alternative hypotheses in the naked goby, we sampled populations across its range and used a phylogeographic approach to test diversification hypotheses in the complex. We used mitochondrial and nuclear DNA markers to examine patterns of genetic diversity and lineage divergence in order to estimate historical gene flow among populations and calculate divergence times among main lineages using coalescence methods. We also used relationships among haplotypes and their relative frequencies to infer the historical demography of different populations of the species across its distribution.

## 2 MATERIALS AND METHODS

### 2.1 Field sampling and DNA sequencing

**Figure 1:**
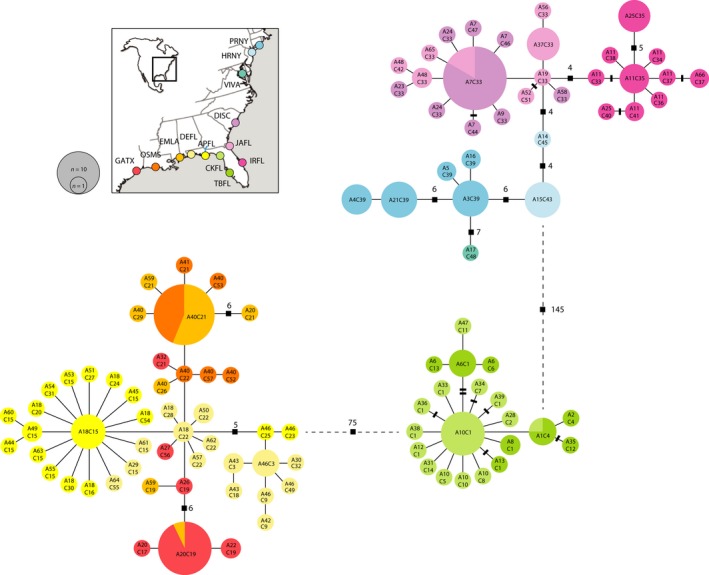
Minimum‐spanning network of 104 unique mtDNA haplotypes obtained from 218 individuals of *Gobiosoma bosc* sampled at 15 localities across its range (see Table 1). Haplotypes shown correspond to concatenated ATPase and COI haplotypes for each individual, each one preceded by “A” or “C,” respectively, in the final haplotype designation. Each circle in the network corresponds to a haplotype, with size proportional to its total frequency. Each branch corresponds to one nucleotide change, and cross‐bars indicate additional changes. Figures next to black squares indicate nucleotide changes between haplotypes when greater than three. Dashed lines indicate the approximate position of branches among the three main phylogroups. Blue line on the map depicts the Apalachicola River

Fish were collected in the field at 15 different localities throughout the species distribution (Figure 1, Table 1) and preserved in 95%–100% ethanol. Genomic DNA was extracted from muscle tissue samples using a Qiagen^™^ DNeasy kit and following the manufacturer's protocol. For the total sample of 218 individuals, we amplified two regions of the mitochondrial genome: an 836‐base pair (bp) region encompassing a fragment of the ATP‐synthase 6 gene and the entire ATP‐synthase 8 gene (hereafter referred to as ATPase) using primers H9236 and L8331 (Perdices, Doadrio, & Bermingham, 2005), and a 613‐bp fragment of the cytochrome *c* oxidase (COI) gene using primers Fish1F and Fish1R (Ward, Zemlak, Innes, Last, & Hebert, 2005). For a smaller subset of 66 individuals from the same localities (see Table 1), we also amplified a 581‐bp fragment of the recombination‐activating gene 1 (RAG1), an autosomal marker, using primers RAG1F and RAG9R (Quenouille, Bermingham, & Planes, 2004). PCR thermal cycles were as follows for the ATPase genes: 2‐min denaturation at 94°C followed by five cycles of 94°C for 45 s, 53°C for 45 s, and 72°C for 90 s, followed by 29 cycles of 94°C for 45 s, 58°C for 45 s, and 72°C for 90 s, and a final extension of 7 min at 72°C; COI gene: 2‐min denaturation at 94°C followed by 35 cycles of 94°C for 30 s, 54°C for 30 s, and 72°C for 60 s, with a final extension of 7 min at 72°C; and RAG1 gene: 5‐min denaturation at 94°C followed by 35 cycles of 94°C for 60 s, 54°C for 60 s, and 72°C for 90 s, with a final extension of 7 min at 72°C. Products were purified with an ethanol precipitation and sequenced in an ABI 3730x automated sequencer. Sequences were automatically aligned using sequencher 4.1 (Gene Codes Corp.), and variable sites were checked visually for accuracy. Sequences were unambiguously translated into their amino acid sequence, and no double peaks were observed in the chromatographs of the mitochondrial sequences, suggesting sequences were of mitochondrial origin and not nuclear copies. All sequences have been deposited in GenBank under the following accessions: MF168974‐MF169100 and MF182408‐182410.

**Table 1:** Sampling localities and sample sizes for mtDNA genes (n_mt_) and the RAG1 nuclear gene (n_nu_)

Locality	Site ID	n_mt_	n_nu_	State	Biogeographic Region	Latitude	Longitude
Hudson River	HRNY	9	4	New York	East Coast	40.622657	−74.068991
Peconic River	PRNY	20	5	New York	East Coast	40.981845	−72.709166
Vims	VIVA	1	1	Virginia	East Coast	37.253843	−76.377703
South Carolina	SC	22	8	S. Carolina	East Coast	32.865721	−79.716628
Jacksonville	JAFL	16	8	Florida	East Coast	30.342613	−81.392674
Indian River	IRFL	18	8	Florida	East FL	27.730127	−80.406792
Rose Bay	RBFL	1	1	Florida	East FL	29.101224	−80.962341
Sebastian River	SRFL	2	1	Florida	East FL	27.863391	−80.488994
Cedar Key	CKFL	24	4	Florida	West FL	29.133051	−83.037926
Tampa Bay	TBFL	14	7	Florida	West FL	27.651607	−82.623163
Apalachicola	APFL	24	4	Florida	Gulf Coast	29.625725	−85.120599
Destin	DEFL	21	1	Florida	Gulf Coast	30.384663	−86.511548
Empire	EMLA	15	4	Louisiana	Gulf Coast	29.308323	−89.533638
Ocean Springs	OSMS	12	3	Mississippi	Gulf Coast	30.401159	−88.83142
Galveston	GATX	19	7	Texas	Gulf Coast	29.338036	−94.724663

### 2.2 Phylogenetic analysis and divergence times

To estimate phylogenetic relationships and divergence times among lineages, we used a coalescence approach that uses Bayesian inference and MCMC simulations to generate posterior probability values for divergence times as implemented in the program beast (Drummond, Suchard, Xie, & Rambaut, 2012). We used jmodeltest (Posada, 2008) to determine the model of sequence evolution for each marker (TrN+G for ATPase and COI, and TrN for RAG1), and we divided our dataset into three gene partitions. Separate analysis of the three partitions gave congruent topologies, so we run a joint analysis of the concatenated markers for a total of 40 unique haplotypes. We used unlinked substitution and clock models for each partition, yet generated a single tree from all three. Our data showed no evidence of clade‐specific rates when using a relaxed‐clock model, so we applied a strict clock model, a Yule speciation model of diversification, and a UPGMA starting tree. After optimizing the priors with preliminary runs, we conducted two final runs of 20 million generations and sampled every 2,000 steps. Chain convergence and burn‐in were assessed with the program tracer v1.5 (Rambaut & Drummond, 2007), and all ESS values in the final runs were above 1,000. As a prior for the ATPase mutation rate, we used 0.0062 changes/site/million years per lineage, which is the average of the rates estimated for *Barbus* (0.0066 c/s/myr, Machordom and Doadrio (2001)), *Prochilodus (*0.0054 c/s/myr, Sivasundar, Bermingham, and Orti(2001)), and geminate Pacific reef fishes (0.0065 c/s/myr, Lessios and Robertson (2006)). We set priors for the other two markers by estimating their rates relative to ATPase (the most variable and informative marker) by running BEAST with uniform prior rates of 1.0, which revealed slower mutation rates for COI and RAG1 by 2.52 and 25.6 times, respectively, in agreement with rates found for these markers in other studies (Perdices et al., 2005; Webb et al., 2004). We set a lognormal distribution for the priors with means of 0.012, 0.005, and 0.0005 for ATPase, COI, and RAG1, respectively, and a log standard deviation of 0.50. As out‐group in all analyses, we used sequences from *Gobiosoma ginsburgi*, which is the closest known relative to *G. bosc* (Rüber et al., 2003).

Traditional bifurcating trees may not adequately represent intraspecific phylogenies, where ancestral and derived haplotypes can coexist in a given sample (Posada & Crandall, 2001), so in order to maximize inference power from haplotype relationships and frequencies, we also constructed minimum‐spanning networks of absolute distances between haplotypes using the molecular‐variance parsimony algorithm (Excoffier & Smouse, 1994) as implemented in arlequin 3.5 (Excoffier & Lischer, 2010).

### 2.3 Historical demography

We calculated haplotype and nucleotide diversity indices for each locality and main regions with dnaSP v5 (Rozas, Sanchez‐DelBarrio, Messeguer, & Rozas, 2003). We tested for past sudden changes in effective population size using Fu's test of neutrality (Fu, 1997), which detects departures from neutrality in scenarios characterized by an excess of rare alleles and young mutations in sequences not subjected to recombination. Significant large negative values of *F*_*s*_ (generated with arlequin 3.5) indicate an excess of recent mutations and reject population stasis (Fu, 1997).

## 3 RESULTS

### 3.1 Genetic variation in mitochondrial and nuclear markers

**Figure 2:**
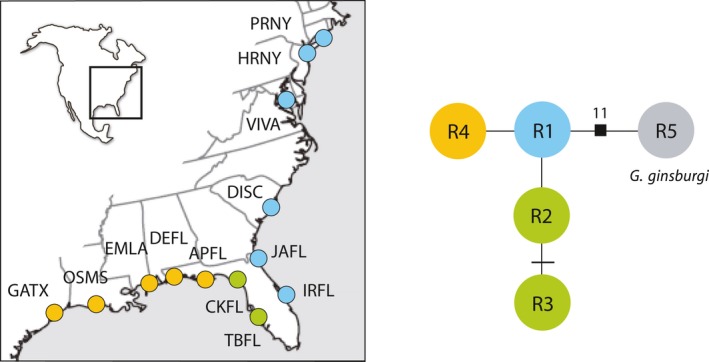
Minimum‐spanning network of four unique RAG1 haplotypes obtained from 62 individuals of *Gobiosoma bosc* sampled at 15 localities (see Table 1). Each circle in the network corresponds to a haplotype. Each branch corresponds to one nucleotide change, and cross‐bars indicate additional changes

Sequencing of 218 individuals from across most of the distribution of *G. bosc* revealed the presence of 66 ATPase haplotypes and 57 COI haplotypes, for a total of 105 unique concatenated mtDNA haplotypes (Table 2). The distribution of mtDNA haplotypes across space was highly structured, and haplotype networks revealed the presence of three main phylogroups corresponding to the Atlantic Coast (from New York to the tip of the Florida peninsula), the west coast of Florida, and the Gulf Coast (Figure 1), as well as additional smaller‐scale structure at the regional level (see below). The 62 individuals sequenced for the autosomal gene RAG1 produced four haplotypes (Figure 2), one found all along the East Coast of North America and including the eastern coast of the Florida peninsula, two restricted to the west coast of Florida, and one found along the Gulf Coast (Figure 2). Within West Florida, all four individuals in the Cedar Key sampling locality (CKFL) were homozygous for allele R2, whereas of the seven individuals sequenced at Tampa Bay (TBFL), three were homozygous for R2, two were homozygous for R3, and two were heterozygous R2/R3.

**Table 2:** MtDNA genetic diversity and population expansion indices, including haplotype diversity (*h*), nucleotide diversity (**π**), and *F*_*s*_ values from Fu's test of neutrality. For population codes, see Table 1

Population	n_mt_	No. mt haps	*h*	SE_*h*_	π	SE_π_	*Fs*
East Coast	68	23	0.8920	0.0239	0.004097	0.002188	−4.122
PRNY	20	5	0.7421	0.0715	0.002473	0.001458	2.636
HRNY	9	2	0.2222	0.1662	0.000598	0.000520	1.844
VIVA	1	1	−	−	−	−	−
SC	22	8	0.5455	0.1276	0.000674	0.000526	−4.744***
JAFL	16	8	0.8417	0.0748	0.001816	0.001141	−1.821
East Florida	21	10	0.8476	0.0588	0.001143	0.000777	−5.249***
IRFL+SRFL+RBFL	21	10	0.8476	0.0588	0.001143	0.000777	−5.249***
E FL+ East Coast	89	33	0.9290	0.0150	0.004436	0.002343	−10.085**
West Florida	38	21	0.9033	0.0379	0.002148	0.001261	−13.484***
TBFL	14	8	0.8901	0.0603	0.002602	0.001560	−1.169
CKFL	24	14	0.8007	0.0867	0.001435	0.000922	−9.523***
Gulf of Mexico	91	49	0.9407	0.0151	0.004673	0.002456	−24.984***
APFL	24	17	0.9203	0.0487	0.001847	0.001131	−13.191***
DEFL	21	15	0.9571	0.0301	0.003657	0.002048	−5.506*
EMLA	15	7	0.6571	0.1384	0.002420	0.001452	−0.203
OSMS	12	6	0.6818	0.1482	0.001042	0.000753	−2.032*
GATX	19	6	0.5380	0.1330	0.002168	0.001308	0.993

*: *p* < 0.05, **: *p* < 0.01, ***: *p* < 0.001.

### 3.2 Genetic structure and divergence at multiple spatial scales within *G. bosc*

**Figure 3:**
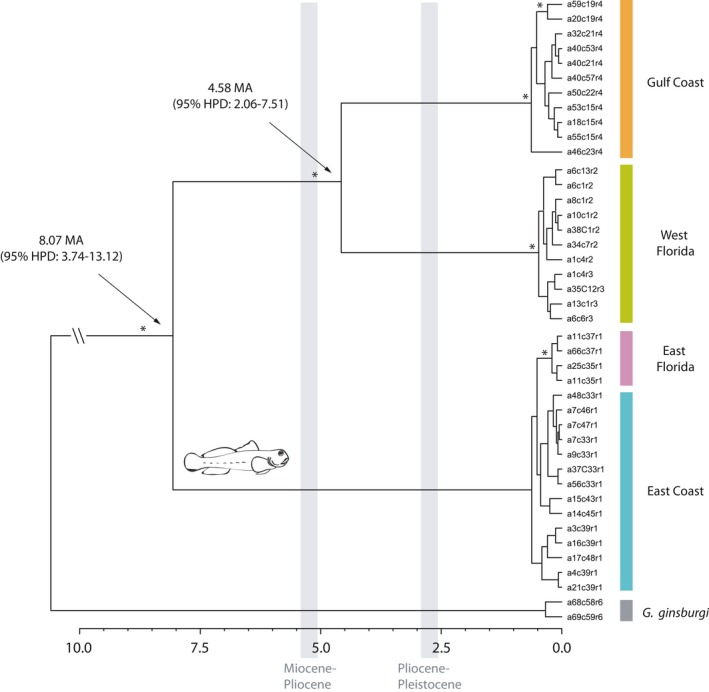
BEAST tree of 40 unique *Gobiosoma bosc* haplotypes obtained by concatenation of ATPase, COI, and RAG1 genes. Asterisks at nodes indicate Bayesian posterior probability of 1. Divergence times for the two main nodes (indicated by arrows) are provided. Time scale at the bottom is in million years

Phylogenetic analysis of mitochondrial and nuclear DNA markers reveals the existence of marked genetic structure within *G. bosc* at various spatial scales. A phylogenetic analysis using Bayesian inference revealed the existence of two major lineages, one corresponding to localities along the Atlantic Coast from New York to the tip of the Florida peninsula and the second one corresponding to West Florida and the Gulf Coast localities (Figure 3). This latter clade is in turn divided into two divergent and well‐supported subclades that correspond, respectively, to the west coast of Florida and all remaining western Gulf Coast localities, from Apalachicola in Florida, to Galveston in Texas (Figure 3). Genetic distances calculated with mtDNA markers and corrected for intrapopulation polymorphism reveal a percent average pairwise distance above 10% between the East Coast and Gulf Coast clades and between East Coast and West Florida clades, and above 5% between Gulf Coast and West Florida clades (Table 3).

**Table 3:** Genetic divergence among populations. Above diagonal: average number of pairwise differences among clades (π_*xy*_); along diagonal: average number of pairwise differences within clades (π_*x*_); below diagonal: percent average pairwise differences among clades corrected for intraclade polymorphism (π_*xy*_ − (π_*x*_ + π_*y*_)/2)

	GC	WFL	EC	EFL
Gulf Coast	6.9	85.3	163.5	162.4
West FL	5.4	3.2	163.9	161.6
East Coast	10.6	10.7	6.1	8.2
East FL	10.6	10.7	0.3	1.7

Mitochondrial DNA haplotype phylogenies reveal further structure within the main three clades (Figures 1 and 3). Along the East Coast, haplotypes sampled at localities along the East Cost of Florida form a distinct phylogroup, which is clearly separated from the rest of the East Coast (Figure 1) and recovered as a clade in the Bayesian inference analysis (Figure 3). Further north along the coast, haplotypes are grouped into three additional geographically structured groups, one corresponding to haplotypes found between Jacksonville, Florida, and the coast of South Carolina, and two corresponding to the two localities sampled in New York (Figure 1). A single sample from Virginia was found to carry a haplotype that is quite divergent from the rest, but whether or not it represents a separate phylogroup will require additional samples. Along the Gulf Coast, there is a weaker geographic pattern of haplotype distribution, and phylogroups are not as site specific as those along the East Coast. For example, most individuals in Galveston, Texas, carry the same or closely related haplotypes, but some individuals were more closely related to phylogroups as far as Destin, Florida, and one individual carrying the most common Galveston haplotype (A20C19) was detected in Louisiana. Finally, no geographic structure was found within the west coast of Florida, with several haplotypes shared between the two localities.

### 3.3 Divergence time among *G. bosc* clades

According to a time divergence analysis using Bayesian inference in a coalescence framework, the separation between the two main clades (Gulf vs. Atlantic) took place about 8.07 MY ago (95% HPD: 3.74–13.12), and the separation between West Florida and the Gulf Coast across the Apalachicola break took place about 4.58 MY ago (95% HPD: 2.06–7.51). In contrast, most of the genetic structure within each of these three main clades originated relatively recently, within the last 500,000 years (Figure 3). Specifically, the clade formed by haplotypes along the eastern coast of Florida diverged from other East Coast populations within the last 200,000 years.

### 3.4 Genetic diversity and historical demography

Patterns of haplotype diversity reveal different demographic histories among *G. bosc* populations. Rapid population expansions that took place in the past can be inferred from “star‐like” patterns in the haplotype networks, where a single high‐frequency haplotype is surrounded by low‐frequency haplotypes one nucleotide change away (Figure 1), and by high negative values of Fu's F_s_ test of neutrality (Table 2). Population expansions appear to have been more prominent in the southern part of the distribution, with highly significant negative *F*_*s*_ values for the Gulf Coast (*F*_*s*_ = −24.98, *p* < 0.0001), and West Florida (*F*_*s*_ = −13.5, *p* < 0.0001), and with localities such as Cedar Key (CKFL) and Apalachicola (APFL) representing clear examples. Diversity patterns in East Florida reject population stasis as well (*F*_*s*_ = −5.25, *p* < 0.0001), yet the rest of the East Coast shows a nonsignificant regional value of *F*_*s*_ (*F*_*s*_ = −4.12, *p* = 0.11), even though the South Carolina locality does show clear signs of expansion (*F*_*s*_ = −4.74, *p* < 0.0001) (Table 2).

## 4 DISCUSSION

### 4.1 Cryptic, independent lineages in the *G. bosc* complex

Despite the lack of apparent phenotypic differentiation, our results reveal that *G. bosc* is composed of three deeply divergent lineages, two of them having diverged several million years ago, and comprising several shallower phylogroups across the range of the complex. The existence of divergent cryptic clades is particularly unexpected in a member of the seven‐spined gobies, a tribe known for striking phenotypic specialization in some geographic regions (Rüber et al., 2003). Our phylogeographic data and estimates of divergence time rule out the hypotheses of recent divergence and extensive gene flow as causes for the lack of phenotypic differentiation within the complex. In contrast, our results seem to support the hypothesis of long‐term phenotypic stasis, with stabilizing selection acting on the ancestral phenotype and preventing differentiation despite the broad ecological gradient occupied by the species along the Atlantic Coast of North America.

The diversification of the seven‐spined gobies is thought to have been driven by major shifts in macrohabitat early in the radiation, followed by more recent adaptive radiations driven by behavioral and niche specialization in different microhabitats at smaller geographic scales (Rüber et al., 2003; Taylor & Hellberg, 2005), and our results suggest that the pattern of lineage divergence within *G. bosc* is somewhat consistent with these two time scales. The main split across the Florida peninsula corresponds to the climatic upheavals of the Miocene/Pliocene boundary, a time of global ecological change with major impact on faunal and floral turnovers (Cerling et al., 1997; LaJeunesse, 2005; Scott, 1997; Webb, 1990), whereas the differentiation of regional phylogroups is likely associated with the glacial cycles during the Pleistocene, which must have had a major impact on estuarine habitats given the marked changes in sea level (Webb, 1990). However, given the broad confidence intervals of our time divergence estimates, these conclusions must be taken with some caution.

The lack of phenotypic divergence associated with the formation of independent lineages in *G. bosc* is striking, both when compared with dramatic radiations in other marine fish groups such as labrid fishes (Alfaro, Brock, Banbury, & Wainwright, 2009), and with other lineages within the Gobiidae (Rüber et al., 2003). Well‐known cases of cryptic divergence in fish include weakfish (Santos, Hrbek, Farias, Schneider, & Sampaio, 2006) and bonefishes of the genus *Albula* (Colborn, Crabtree, Shaklee, Pfeiler, & Bowen, 2001), and among gobies, *G. bosc* is not alone in showing cryptic divergence. In a comprehensive study of the relationship between body shape and diversification rates in Gobiiformes, Thacker (2014) documented an inverse relationship between speciation rate and morphological change in some clades. Cases of cryptic divergence in gobies include the *Gnatholepis thompsoni/scapulostigma* complex, also a seven‐spined goby, which shows a wide geographic distribution across the Atlantic without obvious geographically structured phenotypic variation. However, genetic analysis revealed that the complex has evolved as a result of a relatively recent expansion within the last 150,000 years (Rocha et al., 2005a). Baldwin, Weigt, Smith, and Mounts (2009) showed that in sympatric populations of West Atlantic *Coryphopterus* gobies, sister species pairs that were difficult to tell apart by phenotype belonged to distinct COI lineages divergent by 7.14% (*hyallinus‐personatus*) and 9.51% (*glaucofraenum‐venezuelae*), although the complex is restricted to the temperate zone. The lack of phenotypic differentiation among naked goby populations remains an extreme case of cryptic variation given the large geographic range and pronounced ecological gradient, from subtropical to temperate latitudes.

### 4.2 Phylogeography and historical demography of naked gobies

The main phylogenetic split in the *G. bosc* complex corresponds to the Florida peninsula, a well‐known biogeographic landmark where congruence in contact zones between divergent lineages from the Atlantic and Gulf coasts has been documented for a number of marine organisms, from mollusks to mammals (Adam & Rosel, 2006; Avise, 2000; Soltis, Morris, McLachlan, Manos, & Soltis, 2006). Much more surprising is the deep phylogeographic break found on either side of the Apalachicola River estuary, with over 5% divergence among mtDNA lineages on either side. The Apalachicola River is a well‐known barrier to gene flow for terrestrial organisms (Donovan, Semlitsch, & Routman, 2000; Pauly, Piskurek, & Shaffer, 2007) and freshwater fish (Bagley, Sandel, Travis, Lozano Vilano, & Johnson, 2013; Bermingham & Avise, 1986; Nedbal, Allard, & Honeycutt, 1994; Wooten & Lydeard, 1990), but this is to our knowledge the first reported case for a similar phylogeographic break in a marine fish, although at least one marine mollusk, the arrow squid (*Loligo plei*), was found to show a break there as well (Herke & Foltz, 2002).

The factors that caused the Apalachicola break in naked gobies remains unclear. This is partly because the bathymetry of the region over the Pleistocene was very dynamic, leading to dramatically changing habitat configurations for estuarine species over time (Bagley et al., 2013), and thus affecting species distributions. Freshwater rivers flowing into sea can potentially create barriers to gene flow in coastal species (Rocha, 2003), although adult naked gobies can be found in a broad range of salinities from 0 to 45 parts per thousand (ppt) in estuaries along the Gulf of Mexico (Dawson, 1969). Oligohaline waters appear to be particularly important for larvae, which could restrict gene flow at larger scales. However, the possibility that genetic breaks do not in fact coincide with the barriers that caused them cannot be ruled out at present, and the areas that host phylogeographic breaks may be those where divergent clades come into secondary contact after allopatric differentiation (Craig, Hastings, Pondella, Ross Robertson, & Rosales‐Casián, 2006).

Numerous naked goby populations were characterized by star‐shaped phylogenies of mitochondrial haplotypes, particularly in the eastern Gulf of Mexico and western coast of Florida, indicating sudden population expansions there. Climatic and bathymetric oscillations during the Pleistocene could have caused sudden reductions in estuarine habitats, reducing population sizes and resulting in population bottlenecks that erased genetic diversity through drift. Marked effects of drift have been reported in gobiids at small geographic scales in California (McCraney, Goldsmith, Jacobs, & Kinziger, 2010). Following bottlenecks, population expansions over large geographic areas would have produced the observed genetic patterns. Alternatively, diversity could have been lost during rapid expansions, as drift can reduce diversity at the leading edge of the expansion, without the need to invoke local bottlenecks (Hewitt, 1996). Future work on ecological and microhabitat differences across the phylogeographic barriers documented here will help us understand the relative roles of local adaptation and historical factors in driving lineage divergence in naked gobies.

### 4.3 Taxonomic implications

Cryptic taxa represent a challenge for the discovery and quantification of biodiversity, as their detection requires intensive sampling and costly methods such as phylogenetic analysis or molecular barcoding (Bernardi, 2013; Brickford et al., 2007; von der Heyden et al., 2011; Knowlton, 1993, 2000). Several “cryptic” species of gobies have been described lately (Lima, Freitas, Araujo, & Solé‐Cava, 2005; Neilson & Stepien, 2009; Stepien & Tumeo, 2006; Tang et al., 2010), although in most cases, morphological traits associated with these clades were found a posteriori, following identification of divergent clades using molecular data. Our phylogeographic results revealed three main naked goby lineages with consistent differentiation in the mitochondrial and nuclear genomes, yet no apparent morphological differences. The lack of gene flow among lineages over thousands of generations despite relative geographic proximity suggests that they may have attained reproductive isolation and may deserve species status. Reproductive isolation in fish can evolve fast (Hendry, Wenburg, Volk, & Quinn, 2000), but criteria for species designation typically emphasize diagnosability, so that the detection of phenotypic differences among lineages will likely determine whether a taxonomic revision is warranted. Together with new species being discovered in poorly studied habitats (Tornabene et al., 2016), cryptic taxa may also contribute to the rich diversity of gobies.

## 5 CONCLUSIONS

Contrasting with the general pattern observed in seven‐spined gobies as a group, where ecology was seen to play a more important role than biogeography in species diversification, our results suggest that in some taxa like the naked goby, geography and drift were more important than ecology and selection in differentiating populations. The lack of phenotypic divergence despite marked genetic structure in neutral markers at different spatial scales suggests that stabilizing selection has prevented the ancestral phenotype from differentiating despite the broad environmental and latitudinal range occupied by the species.

## CONFLICT OF INTEREST

None declared.
